# $\nu$-Improved nonparallel support vector machine

**DOI:** 10.1038/s41598-022-22559-5

**Published:** 2022-10-25

**Authors:** Fengmin Sun, Shujun Lian

**Affiliations:** School of Management Science, Qufu Normal University, Rizhao, China

**Keywords:** Engineering, Mathematics and computing

## Abstract

In this paper, a $\nu$-improved nonparallel support vector machine ($\nu$-IMNPSVM) is proposed to solve binary classification problems. In this model, we use related ideas of $\nu$-support vector machine($\nu$-SVM), the parameter $\nu$ is introduced to control the limits of the support vectors percentage. In the objective function, the parameter $\varepsilon$ is increased to ensure that $\varepsilon$-band is kept as small as possible. It has played a great role in the classification of unbalanced data sets. On the basis of maximizing the interval between two classes, $\nu$-IMNPSVM can fully fit the distribution of data points in the class by minimizing the $\varepsilon$-band, which enhances the generalization ability of the model. The results on the benchmark datasets testify that the proposed model has a good effect on the classification accuracy.

## Introduction

Support vector machines (SVM), introduced by Vapnik in the early 1990s, is a helpful tool for pattern recognition and logistic regression^1–4^. Due to its three elements: interval maximization, duality theory, and nuclear techniques, it has been used in diversified fields, including face recognition, text classification and bioinformatics^5–9^. SVM adopts the principle of structural risk minimization, which not only maximizes the classification interval, but also keeps the error small. In traditional support vector machines, two parallel support hyperplanes are constructed through a quadratic programming problem(QPP). It ensures maximum interval between hyperplanes and takes the intermediate hyperplane as the final decision hyperplane.

In recent years, in order to obtain higher classification accuracy in shorter training time, scholars have made a lot of efforts in this regard. The research on support vector machine generally includes: from the perspective of algorithm, the training speed is improved by optimizing the algorithm, such as sequential minimal optimization (SMO)^10^, SSSR^11^, incremental learning^12^ and so on. From the perspective of the model, the original problem is partially adjusted to obtain higher classification accuracy and faster classification speed, such as MVSVM^13^, PPSVC^14^, NHSVM^15^, and so on^16–25^. Most of the research is based on the concept of nonparallel classifier.

So far, nonparallel hyperplane classifiers have caused wide concern among scholars. Mangasarian and Wild^16^ first proposed generalized eigenvalue proximal support vector machine (GEPSVM). Two kinds of hyperplanes are constructed by solving the eigenvectors corresponding to the minimum eigenvalues of two related generalized eigenvalue problems, so that each hyperplane is as close as possible to one class in the classification samples. Then Jayadeva et al.^17^ proposed twin support vector machine (TWSVM). It follows the thinking of GEPSVM to get two nonparallel hyperplanes by solving two smaller quadratic programming problems (QPPs), which is about 4 times faster than the training speed of SVM. Based on TWSVM, twin bounded support vector machine (TBSVM) was presented in Ref.^18^. It was further developed in Ref.^19–23^. In order to facilitate the application of kernel techniques in dual problems, Tian et al.^24^ proposed nonparallel support vector machine (NPSVM), which further improved the nonparallel hyperplane classifier. For NPSVM, it also looks for two nonparallel hyperplanes. Two $\varepsilon$-bands are constructed on either side of each hyperplane, and each $\varepsilon$-band is guaranteed to contain a class of points as much as possible. Each class of point maintains at least one distance from the other hyperplane. Because the penalty parameter *C* lacks practical significance, the sparsity of the model cannot be estimated even if the value of *C* is given. So $\nu$-nonparallel support vector machine ($\nu$-NPSVM) was further proposed in Ref.^25^. It combines $\nu$-SVM and $\nu$-support vector regression($\nu$-SVR) to obtain two nonparallel hyperplanes. The value of $\nu$ replaces the position of parameter *C*, and the number of support vectors can be controlled by adjusting parameter $\nu$. To solve the difficulty of large-scale data set processing, DC-$\nu$NPSVM was subsequently proposed in Ref.^26^. It makes $\nu$-NPSVM have faster convergence speed and even higher classification accuracy when processing large-scale data sets.

Based on the previous model, in this paper, we propose a new support vector machine, called $\nu$-improved nonparallel support vector machine ($\nu$-IMNPSVM). It optimizes the model of NPSVM and minimizes the constructed $\varepsilon$-band. In order to overcome the lack of quantitative meaning of parameters, the value of $\nu$ is introduced in combination with the characteristics of $\nu$-SVM. It enables the sparsity of the model to be presented, thus inheriting the advantages of $\nu$-SVM and making the number of support vectors easy to control. $\nu$-IMNPSVM, meanwhile, inherits the advantages of the previous nonparallel classifier and improves the running speed. Compared with $\nu$-NPSVM and DC-$\nu$NPSVM, $\nu$-IMNPSVM adopts a different concept. It only applies the idea of $\nu$-SVM without considering $\nu$-SVR. we replace the position of *C* with parameter $\nu$ and retain the free parameter $\varepsilon$. Moreover, the regularization term is added to ensure the uniqueness of the decision function. The classification accuracy is further improved by optimizing and adjusting the $\varepsilon$-band.

This paper is organized as follows. In “Background” section, we briefly introduce standard *C*-SVM, $\nu$-SVM, NPSVM and $\nu$-NPSVM. In “[Sec Sec7]” section, we propose our new support vector machine, termed $\nu$-IMNPSVM. In “Experimental result” section, we deal with experimental results. In “Conclusion” section, concluding remarks are given.

## Background

In this section, we briefly introduce standard *C*-SVM, $\nu$-SVM, NPSVM and $\nu$-NPSVM.

### *C*-SVM

Consider the binary classification problem with the training set

1 $$\begin{aligned} T=\bigg \{(x_{1},y_{1}),\ldots ,(x_{l},y_{l})\bigg \}, \end{aligned}$$

where *l* is the number of samples, $x_{i}\in \mathcal {R}^{n}$ are inputs, $y_{i}\in \{1,-1\},i=1,\ldots ,l$ are the labels, the standard *C*-SVM formulates the problem as a convex QPP:

2 $$\begin{aligned} \begin{array}{ll} &{}\min \limits _{\omega ,b,\xi }\displaystyle \frac{1}{2}\parallel \omega \parallel ^{2}+C \sum \limits _{i=1}^{l}\xi _{i}\\ &{}\text{ s.t. }~~y_{i}((\omega \cdot x_{i})+b)\ge 1-\xi _{i},~~~~~~i=1,\ldots ,l,\\ &{} \qquad \xi _{i}\ge 0,~~~~~i=1,\ldots ,l,\\ \end{array} \end{aligned}$$

where $C>0$ is a penalty parameter, $\xi _{i}=(\xi _{1},\ldots ,\xi _{l})^\text {T}$. The importance of minimizing training errors and maximizing intervals can be balanced by adjusting the value of *C*.

The separation hyperplane of *C*-SVM is

3 $$\begin{aligned} (\omega \cdot x)+b=0. \end{aligned}$$

The detached hyperplane lies midway between $(\omega \cdot x)+b=-1$ and $(\omega \cdot x)+b=1$. For new input $x\in R^{n}$, we can determine which category it belongs to by using the following decision function

4 $$\begin{aligned} f(x)=sgn((\omega \cdot x)+b). \end{aligned}$$

### $\nu$-SVM

Consider the binary classification problem with the training set (1), $\nu$-SVM formulates the problem as a convex QPP:

5 $$\begin{aligned} \begin{array}{ll} &{}\min \limits _{\omega , b, \xi , \rho }\displaystyle \frac{1}{2}\parallel \omega \parallel ^{2}-\nu \rho +\frac{1}{l}\sum \limits _{i=1}^{l}\xi _{i}\\ &{}\text{ s.t. }~~ y_{i}((\omega \cdot x_{i})+b)\ge \rho -\xi _{i},~~i=1,\ldots ,l,\\ &{} \qquad \xi _{i}\ge 0,~~~~~i=1,\ldots ,l,\\ &{} \qquad \rho \ge 0,\\ \end{array} \end{aligned}$$

where $\xi _{i}=(\xi _{1},\ldots ,\xi _{l})^\text {T}$, and $\nu \in (0,1]$. The value of $\nu$ has real meaning. If the number of support vectors is *q* and the total number of sample points is *l*, there is always $\frac{q}{l}\ge \nu$.

### NPSVM

For the binary classification problem with the training set

6 $$\begin{aligned} T=\bigg \{(x_{1},+1),\ldots ,(x_{p},+1),(x_{p+1},-1),\ldots ,(x_{p+q},-1)\bigg \}, \end{aligned}$$

where $x_{i}\in \mathcal {R}^{n},i=1,\ldots ,p+q$. NPSVM also seeks two nonparallel hyperplanes

7 $$\begin{aligned} (\omega _{+}\cdot x)+b_{+}=0 ~\hbox {and}~ (\omega _{-}\cdot x)+b_{-}=0, \end{aligned}$$

by solving the following two QPPs:

8 $$\begin{aligned} \begin{array}{ll} &{}\min \limits _{\omega _{+},b_{+},\eta _{+}^{(*)}\xi _{-}}\displaystyle \frac{1}{2}\parallel \omega _{+}\parallel ^{2}+C_{1}\sum \limits _{i=1}^{p}(\eta _{i}+\eta _{i}^{*})+C_{2}\sum \limits _{j=p+1}^{p+q}\xi _{j}\\ &{} ~~\text{ s.t. }~ (\omega _{+}\cdot x_{i})+b_{+}\le \varepsilon +\eta _{i},~~i=1,\ldots ,p,\\ &{} \qquad -(\omega _{+}\cdot x_{i})-b_{+}\le \varepsilon +\eta _{i}^{*},~~i=1,\ldots ,p,\\ &{} \qquad (\omega _{+}\cdot x_{j})+b_{+}\le -1+\xi _{j},~~j=p+1,\ldots ,p+q,\\ &{} \qquad \eta _{i},\eta _{i}^{*}\ge 0,~~i=1,\ldots ,p,\\ &{} \qquad \xi _{j}\ge 0,~~j=p+1,\ldots ,p+q,\\ \end{array} \end{aligned}$$

and

9 $$\begin{aligned} \begin{array}{ll} &{}\min \limits _{\omega _{-},b_{-},\eta _{-}^{(*)}\xi _{+}}\displaystyle \frac{1}{2}\parallel \omega _{-}\parallel ^{2}+C_{3}\sum \limits _{i=p+1}^{p+q}(\eta _{i}+\eta _{i}^{*})+C_{4}\sum \limits _{j=1}^{p}\xi _{j}\\ &{}\text{ s.t. }~~ (\omega _{-}\cdot x_{i})+b_{-}\le \varepsilon +\eta _{i},~~i=p+1,\ldots ,p+q,\\ &{} \quad -(\omega _{-}\cdot x_{i})-b_{-}\le \varepsilon +\eta _{i}^{*},~~i=p+1,\ldots ,p+q,\\ &{} \quad (\omega _{-}\cdot x_{j})+b_{-}\ge 1-\xi _{j},~~j=1,\ldots ,p,\\ &{} \quad \eta _{i},\eta _{i}^{*}\ge 0,~~i=p+1,\ldots ,p+q,\\ &{} \quad \xi _{j}\ge 0,~~j=1,\ldots ,p,\\ \end{array} \end{aligned}$$

where $x_{i},i=1,\ldots ,p$ are positive inputs, and $x_{i},i=p+1,\ldots ,p+q$ are negative inputs, $C_{i}\ge 0, i=1,\ldots ,4$ are penalty parameters. $\xi _{+}=(\xi _{1},\ldots ,\xi _{p})^\text {{T}}$, $\xi _{-}=(\xi _{p+1},\ldots ,\xi _{p+q})^\text {{T}}$, $\eta _{+}^{(*)}=(\eta _{+}^\text {{T}},\eta _{+}^{*\text {T}})^\text {{T}}=(\eta _{1},\ldots ,\eta _{p},\eta _{1}^{*},\ldots ,\eta _{p}^{*})^\text {{T}}$, $\eta _{-}^{(*)}=(\eta _{-}^\text {{T}},\eta _{-}^{*\text {T}})^\text {{T}}=(\eta _{p+1},\ldots ,\eta _{p+q},\eta _{p+1}^{*},\ldots ,\eta _{p+q}^{*})^\text {{T}}$ are slack variables.

When appropriate parameters are selected, NPSVM degrades to TWSVM.

### $\nu$-NPSVM

$\nu$-NPSVM is based on NPSVM, combining $\nu$-SVM and $\nu$-SVR and inheriting all their advantages. It find two nonparallel hyperplanes by solving the following two convex QPPs:

10 $$\begin{aligned} \begin{array}{ll} &{}\min \limits _{\omega _{+},b_{+},\eta _{+}^{*}\xi _{-},\rho _{+},\varepsilon _{+}}\displaystyle \frac{1}{2}\parallel \omega _{+}\parallel ^{2}+C_{1}\bigg (\nu _{1}\varepsilon _{+}+\frac{1}{p}\sum \limits _{i=1}^{p}(\eta _{i}+\eta _{i}^{*})\bigg )\\ &{} \qquad \quad +\bigg (-\nu _{2}\rho _{+}+\frac{1}{q}\sum \limits _{j=p+1}^{p+q}\xi _{j}\bigg )\\ &{}\text{ s.t. }~~ (\omega _{+}\cdot x_{i})+b_{+}\le \varepsilon _{+}+\eta _{i},~~i=1,\ldots ,p,\\ &{} \qquad -(\omega _{+}\cdot x_{i})-b_{+}\le \varepsilon _{+}+\eta _{i}^{*},~~i=1,\ldots ,p,\\ &{} \qquad (\omega _{+}\cdot x_{j})+b_{+}\le -\rho _{+}+\xi _{j},~~j=p+1,\ldots ,p+q,\\ &{} \qquad \eta _{i},\eta _{i}^{*}\ge 0,~~i=1,\ldots ,p,\\ &{} \qquad \xi _{j}\ge 0,~~j=p+1,\ldots ,p+q,\\ &{} \qquad \rho _{+}\ge 0,\varepsilon _{+}\ge 0,\\ \end{array} \end{aligned}$$

and

11 $$\begin{aligned} \begin{array}{ll} &{}\min \limits _{\omega _{-},b_{-},\eta _{-}^{*}\xi _{+},\rho _{-},\varepsilon _{-}}\displaystyle \frac{1}{2}\parallel \omega _{-}\parallel ^{2}+C_{3}(\nu _{3}\varepsilon _{-}+\frac{1}{q}\sum \limits _{i=p+1}^{p+q}(\eta _{i}+\eta _{i}^{*}))\\ &{} \qquad +\bigg (-\nu _{4}\rho _{-}+\frac{1}{p}\sum \limits _{j=1}^{p}\xi _{j}\bigg )\\ &{}\text{ s.t. }~~ (\omega _{-}\cdot x_{i})+b_{-}\le \varepsilon _{-}+\eta _{i},~~i=p+1,\ldots ,p+q,\\ &{} \qquad -(\omega _{-}\cdot x_{i})-b_{-}\le \varepsilon _{-}+\eta _{i}^{*},~~i=p+1,\ldots ,p+q,\\ &{} \qquad (\omega _{-}\cdot x_{j})+b_{-}\ge \rho _{-}-\xi _{j},~~j=1,\ldots ,p,\\ &{} \qquad \eta _{i},\eta _{i}^{*}\ge 0,~~i=p+1,\ldots ,p+q,\\ &{} \qquad \xi _{j}\ge 0,~~j=1,\ldots ,p,\\ &{} \qquad \rho _{-}\ge 0,\varepsilon _{-}\ge 0,\\ \end{array}\end{aligned}$$

where $x_{i},i=1,\ldots ,p$ are positive inputs, and $x_{i},i=p+1,\ldots ,p+q$ are negative inputs, $C_{i}\ge 0, i=1,\ldots ,4, \nu _{i}\in (0,1], i=1, \dots ,4$ are penalty parameters. $\xi _{+}=(\xi _{1},\ldots ,\xi _{p})^{T}$, $\xi _{-}=(\xi _{p+1},\ldots ,\xi _{p+q})^\text {{T}}$, $\eta _{+}^{(*)}=(\eta _{+}^\text {{T}},\eta _{+}^{*\text {T}})^\text {{T}}=(\eta _{1},\ldots ,\eta _{p},\eta _{1}^{*},\ldots ,\eta _{p}^{*})^\text {{T}}$, $\eta _{-}^{(*)}=(\eta _{-}^\text {{T}},\eta _{-}^{*\text {T}})^\text {{T}}=(\eta _{p+1},\ldots ,\eta _{p+q},\eta _{p+1}^{*},\ldots ,\eta _{p+q}^{*})^\text {{T}}$ are slack variables.

## $\nu$-IMNPSVM

In this section, we propose a new nonparallel support vector machine, called $\nu$-improved nonparallel support vector machine.

### Linear $\nu$-IMNPSVM

#### Primal problems

We seek the two nonparallel hyperplanes (7) by solving two convex QPPs:

12 $$\begin{aligned} \begin{array}{ll} &{}\min \limits _{\omega _{+},b_{+},\varepsilon _{+},\eta _{+}^{(*)},\rho _{+},\xi _{-}}~~ \displaystyle \frac{1}{2}(\Vert \omega _{+}\Vert ^{2}+b_{+}^{2})+C_{1}\varepsilon _{+}+C_{2}\sum \limits _{i=1}^{p}(\eta _{i}+\eta _{i}^{*})\\ &{} \qquad \quad + \bigg (-\nu _{1}\rho _{+}+\frac{1}{q}\sum \limits _{j=p+1}^{p+q}\xi _{j}\bigg ) \\ &{}\text{ s.t. }~~ (\omega _{+}\cdot x_{i})+b_{+}\le \varepsilon _{+}+\eta _{i},i=1,\ldots ,p,\\ &{} \qquad -(\omega _{+}\cdot x_{i})-b_{+}\le \varepsilon _{+}+\eta _{i}^{*},i=1,\ldots ,p,\\ &{} \qquad (\omega _{+}\cdot x_{j})+b_{+}\le -\rho _{+}+\xi _{j},j=p+1,\ldots ,p+q,\\ &{} \qquad \eta _{i},\eta _{i}^{*}\ge 0,i=1,\ldots ,p,\\ &{} \qquad \xi _{j}\ge 0,j=p+1,\ldots ,p+q,\\ &{} \qquad \varepsilon _{+}\ge 0,\rho _{+}\ge 0,\\ \end{array} \end{aligned}$$

and

13 $$\begin{aligned} \begin{array}{ll} &{}\min \limits _{\omega _{-},b_{-},\varepsilon _{-},\eta _{-}^{(*)},\rho _{-},\xi _{+}}~~ \displaystyle \frac{1}{2}(\Vert \omega _{-}\Vert ^{2}+b_{-}^{2})+C_{3}\varepsilon _{-}+C_{4}\sum \limits _{j=p+1}^{p+q}\bigg (\eta _{j}+\eta _{j}^{*}\bigg )\\ &{} \qquad \quad +\bigg (-\nu _{2}\rho _{-}+\frac{1}{p}\sum \limits _{i=1}^{p}\xi _{i}\bigg ) \\ &{}\text{ s.t. }~~ (\omega _{-}\cdot x_{j})+b_{-}\le \varepsilon _{-}+\eta _{j},j=p+1,\ldots ,p+q,\\ &{} \qquad -(\omega _{-}\cdot x_{j})-b_{-}\le \varepsilon _{-}+\eta _{j}^{*},j=p+1,\ldots ,p+q,\\ &{} \qquad (\omega _{-}\cdot x_{i})+b_{-}\ge \rho _{-}-\xi _{i},i=1,\ldots ,p,\\ &{} \qquad \eta _{j},\eta _{j}^{*}\ge 0,j=p+1,\ldots ,p+q,\\ &{} \qquad \xi _{i}\ge 0,i=1,\ldots ,p,\\ &{} \qquad \varepsilon _{-}\ge 0,\rho _{-}\ge 0,\\ \end{array}\end{aligned}$$

where $x_{i},i=1,\ldots ,p$ are positive inputs, and $x_{j}, j=p+1,\ldots ,p+q$ are negative inputs, $C_{i}\ge 0, i=1,2,3,4$, $\nu _{i}\in (0,1], i=1,2$ are parameters. $\xi _{+}=(\xi _{1},\ldots ,\xi _{p})^{T}$, $\xi _{-}=(\xi _{p+1},\ldots ,\xi _{p+q})^{T}$, $\eta _{+}^{(*)}=(\eta _{+}^{T},\eta _{+}^{*T})^{T}=(\eta _{1},\ldots ,\eta _{p},\eta _{1}^{*},\ldots ,\eta _{p}^{*})^{T}$, $\eta _{-}^{(*)}=(\eta _{-}^{T},\eta _{-}^{*T})^{T}=(\eta _{p+1},\ldots ,\eta _{p+q},\eta _{p+1}^{*},\ldots ,\eta _{p+q}^{*})^{T}$ are slack variables.

There are four parts in the objective function (12) or (13). The first part is the regular term to minimize structural risk. The second part adds constraint variables to the constructed $\varepsilon$-band to keep it as small as possible. The third and fourth parts ensure that the training error is as small as possible, and that the distance from two hyperplanes to points in the other class is as far as possible.

**Figure 1:**
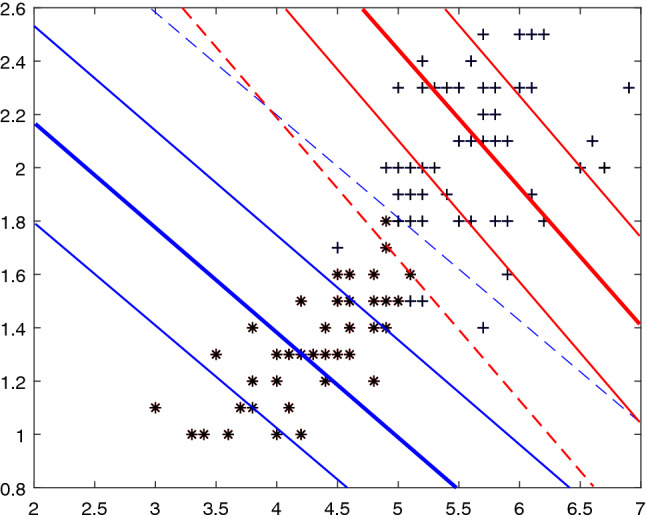
Geometrical illustration of problem (12) in $R^{2}$.

Now, we discuss the primal problem (12) geometrically in $R^{2}$ (see Fig. 1). Firstly, We hope that the class point +s fell as much as possible in hyperplane $(\omega _{+}\cdot x)+b_{+}=\varepsilon$ and $(\omega _{+}\cdot x)+b_{+}=-\varepsilon$ (red thin solid line) within the $\varepsilon$-band, and the $\varepsilon$-band is as small as possible. Secondly, we want the negative class point $*$s to be as far away from the hyperplane $(\omega _{+}\cdot x)+b_{+}=-\rho _{+}$ (red dotted line).

#### Dual problems of (12) and (13)

Now, in order to get the solutions of problems (12) and (13), we need to derive their dual problems. For the above QPP (12), we introduce its Lagrangian function

14 $$\begin{aligned} \begin{array}{ll} &{}L\bigg (\omega _{+},b_{+},\varepsilon _{+},\eta _{i},\eta _{i}^{*},\rho _{+},\xi _{j},\alpha _{i},\alpha _{i}^{*},\beta _{j},\gamma _{i},\gamma _{i}^{*},\lambda _{j},\theta _{1},\theta _{2}\bigg )\\ &{}\quad =\displaystyle \frac{1}{2}\bigg (\parallel \omega _{+}\parallel ^2+b_{+}^2\bigg )+C_{1}\varepsilon _{+}+C_{2}\sum \limits _{i=1}^{p}\bigg (\eta _{i}+\eta _{i}^{*}\bigg )-\nu _{1}\rho _{+}+\frac{1}{q}\sum \limits _{j=p+1}^{p+q}\xi _{j}\\ &{}\qquad +\sum \limits _{i=1}^{p}\alpha _{i}\bigg ((\omega _{+}\cdot x_{i})+b_{+}-\varepsilon _{+}-\eta _{i}\bigg )+\sum \limits _{i=1}^{p}\alpha _{i}^{*}\bigg (-(\omega _{+}\cdot x_{i})-b_{+}-\varepsilon _{+}-\eta _{i}^{*}\bigg )\\ &{}\qquad +\sum \limits _{j=p+1}^{p+q}\beta _{j}\bigg ((\omega _{+}\cdot x_{j})+b_{+}+\rho _{+}-\xi _{j}\bigg )-\sum \limits _{i=1}^{p}\gamma _{i}\eta _{i}-\sum \limits _{i=1}^{p}\gamma _{i}^{*}\eta _{i}^{*}-\sum \limits _{j=p+1}^{p+q}\lambda _{j}\xi _{j}\\ &{}\qquad -\, \theta _{1}\varepsilon _{+}-\theta _{2}\rho _{+},\\ \end{array} \end{aligned}$$

where $\alpha _{+}=(\alpha _{1},\ldots ,\alpha _{p})^\text {T}, \alpha _{+}^{*}=(\alpha _{1}^{*},\ldots ,\alpha _{p}^{*})^\text {T}, \beta _{-}=(\beta _{p+1},\ldots ,\beta _{p+q})^\text {T}, \gamma _{+}=(\gamma _{1},\ldots ,\gamma _{p})^\text {T}, \gamma _{+}^{*}=(\gamma _{1}^{*},\ldots ,\gamma _{p}^{*})^\text {T}, \lambda _{-}=(\lambda _{p+1},\ldots ,\lambda _{p+q})^\text {T}, \theta _{1}\in R, \theta _{2} \in R$ are Lagrange multiplier vectors.

For $\omega _{+}, b_{+},\varepsilon _{+},\eta _{+},\eta _{+}^{*},\rho _{+},\xi _{-}$, we use *Karush*–*Kuhn*–*Tucker*(KKT) conditions^27^ and get the following relationships:

15 $$\begin{aligned}&\nabla _{\omega _{+}}L=\omega _{+}+\sum _{i=1}^{p}\alpha _{i}x_{i}-\sum _{i=1}^{p}\alpha _{i}^{*}x_{i}+\sum _{j=p+1}^{p+q}\beta _{j}x_{j}=0, \end{aligned}$$

16 $$\begin{aligned}&\nabla _{b_{+}}L=b_{+}+\sum _{i=1}^{p}\alpha _{i}-\sum _{i=1}^{p}\alpha _{i}^{*}+\sum _{j=p+1}^{p+q}\beta _{j}=0,\end{aligned}$$

17 $$\begin{aligned}&\nabla _{\varepsilon _{+}}L=C_{1}-\sum _{i=1}^{p}\alpha _{i}-\sum _{i=1}^{p}\alpha _{i}^{*}-\theta _{1}=0, \end{aligned}$$

18 $$\begin{aligned}&\nabla _{\eta _{+}}L=C_{2}-\sum _{i=1}^{p}\alpha _{i}-\sum _{i=1}^{p}\gamma _{i}=0,\end{aligned}$$

19 $$\begin{aligned}&\nabla _{\eta _{+}^{*}}L=C_{2}-\sum _{i=1}^{p}\alpha _{i}^{*}-\sum _{i=1}^{p}\gamma _{i}^{*}=0,\end{aligned}$$

20 $$\begin{aligned}&\nabla _{\rho _{+}}L=-\nu _{1}+\sum _{j=p+1}^{p+q}\beta _{j}-\theta _{2}=0,\end{aligned}$$

21 $$\begin{aligned}&\nabla _{\xi _{-}}L=\frac{1}{q}-\sum _{j=p+1}^{p+q}\beta _{j}-\sum _{j=p+1}^{p+q}\lambda _{j}=0, \end{aligned}$$

22 $$\begin{aligned}&(\omega _{+}\cdot x_{i})+b_{+}\le \varepsilon _{+}+\eta _{i}, \eta _{i}\ge 0,i=1,\ldots ,p,\end{aligned}$$

23 $$\begin{aligned}&-(\omega _{+}\cdot x_{i})-b_{+}\le \varepsilon _{+}+\eta _{i}^{*}, \eta _{i}^{*}\ge 0,i=1,\ldots ,p,\end{aligned}$$

24 $$\begin{aligned}&(\omega _{+}\cdot x_{j})+b_{+}\le -\rho _{+}+\xi _{j}, \xi _{j}\ge 0,j=p+1,\ldots ,p+q,\end{aligned}$$

25 $$\begin{aligned}&\alpha _{i},\alpha _{i}^{*},\gamma _{i},\gamma _{i}^{*}\ge 0,i=1,\ldots ,p,\end{aligned}$$

26 $$\begin{aligned}&\beta _{j},\lambda _{j}\ge 0,j=p+1,\ldots ,p+q,\end{aligned}$$

27 $$\begin{aligned}&\theta _{1},\theta _{2}\ge 0. \end{aligned}$$

From (17), (20) and (27), we get

28 $$\begin{aligned}&C_{1}-\sum _{i=1}^{p}\alpha _{i}-\sum _{i=1}^{p}\alpha _{i}^{*}\ge 0,\end{aligned}$$

29 $$\begin{aligned}&-\nu _{1}+\sum _{j=p+1}^{p+q}\beta _{j}\ge 0. \end{aligned}$$

From (18), (19), (25) and (26), we get

30 $$\begin{aligned}&0\le \alpha _{+},\alpha _{+}^{*}\le C_{2}e_{+},\end{aligned}$$

31 $$\begin{aligned}&0\le \beta _{-}\le \frac{1}{q}e_{-}. \end{aligned}$$

From (15) and (16), we get

32 $$\begin{aligned} \omega _{+}= & {} \sum _{i=1}^{p}(\alpha _{i}^{*}-\alpha _{i})x_{i}-\sum _{j=p+1}^{p+q}\beta _{j}x_{j},\end{aligned}$$

33 $$\begin{aligned} b_{+}= & {} \sum _{i=1}^{p}(\alpha _{i}^{*}-\alpha _{i})-\sum _{j=p+1}^{p+q}\beta _{j}.~~~~~ \end{aligned}$$

Then putting (32) and (33) into the Lagrangian function (14), and using (15)–(21), we get dual problem of (12) as follows:

34 $$\begin{aligned} \begin{array}{ll} \min \limits _{\alpha _{+},\alpha _{+}^{*},\beta _{-}}~&{}\displaystyle \frac{1}{2}\sum _{i=1}^{p}\sum _{j=1}^{p}(\alpha _{i}^{*}-\alpha _{i})(\alpha _{j}^{*}-\alpha _{j})((x_{i}\cdot x_{j})+1)\\ &{}\quad -\sum \limits _{i=1}^{p}\sum \limits _{j=p+1}^{p+q}(\alpha _{i}^{*}-\alpha _{i})\beta _{j}((x_{i}\cdot x_{j})+1)\\ &{}\quad +\displaystyle \frac{1}{2}\sum \limits _{i=p+1}^{p+q}\sum \limits _{j=p+1}^{p+q}\beta _{i}\beta _{j}((x_{i}\cdot x_{j})+1)\\ \text{ s.t. }~&{}C_{1}-\sum \limits _{i=1}^{p}\alpha _{i}-\sum \limits _{i=i}^{p}\alpha _{i}^{*}\ge 0,\\ &{}\quad -\nu _{1}+\sum \limits _{j=p+1}^{p+q}\beta _{j}\ge 0,\\ &{}\quad 0\le \alpha _{i},\alpha _{i}^{*}\le C_{2},~~ i=1,\cdots ,p,\\ &{}\quad 0\le \beta _{j}\le \frac{1}{q},~~j=p+1,\cdots ,p+q.\\ \end{array} \end{aligned}$$

Equation (34) is equal to the following QPP:

35 $$\begin{aligned} \begin{array}{ll} &{}\min \limits _{\pi }~\displaystyle \frac{1}{2}\pi ^\text {T}\Lambda \pi \\ &{}\text{ s.t. }~-\nu _{1}+\delta ^\text {T}\pi \ge 0,\\ &{}\quad C_{1}-\psi ^\text {T}\pi \ge 0,\\ &{}\quad 0\le \pi \le C,\\ \end{array} \end{aligned}$$

where

$$\begin{aligned} \pi= & {} ({\alpha _{+}^{*}}^\text {T},\alpha _{+}^\text {T},\beta _{-}^\text {T})^\text {T},\\ \delta= & {} (0e_{+}^{\text {T}},0e_{+}^{\text {T}},e_{-}^{\text {T}})^\text {T},\\ \psi= & {} (e_{+}^\text {T},e_{+}^\text {T},0e_{-}^\text {T})^\text {T},\\ C= & {} \bigg (C_{2}e_{+}^\text {T},C_{2}e_{+}^\text {T},\frac{1}{q}e_{-}^\text {T}\bigg )^\text {T}, \end{aligned}$$

and

$$\begin{aligned} \begin{aligned}\Lambda&=\left( \begin{array}{ll} \Lambda _{1} &{} -\Lambda _{2} \\ -\Lambda _{2}^\text {T} &{} ~~\Lambda _{3} \\ \end{array} \right) ,\\ \Lambda _{1}&=\left( \begin{array}{ll} AA^\text {T}+e_{+}e_{+}^\text {T} &{} -(AA^\text {T}+e_{+}e_{+}^\text {T})\\ -(AA^\text {T}+e_{+}e_{+}^\text {T}) &{} ~~AA^\text {T}+e_{+}e_{+}^\text {T}\\ \end{array} \right) ,~\\ \Lambda _{2}&=\left( \begin{array}{ll} AB^\text {T}+e_{+}e_{-}^\text {T} \\ -(AB^\text {T}+e_{+}e_{-}^\text {T}) \\ \end{array} \right) , \Lambda _{3}=BB^\text {T}+e_{-}e_{-}^\text {T}. \end{aligned} \end{aligned}$$

In the same way, the dual of the problem (13) is as follows:

36 $$\begin{aligned} \begin{array}{ll} \min \limits _{\alpha _{-},\alpha _{-}^{*},\beta _{+}}&{}~~\displaystyle \frac{1}{2}\sum \limits _{i=p+1}^{p+q}\sum \limits _{j=p+1}^{p+q}(\alpha _{i}^{*}-\alpha _{i})(\alpha _{j}^{*}-\alpha _{j})((x_{i}\cdot x_{j})+1)\\ &{}\quad -\sum \limits _{j=1}^{p}\sum \limits _{i=p+1}^{p+q}(\alpha _{i}^{*}-\alpha _{i})\beta _{j}((x_{i}\cdot x_{j})+1)\\ &{}\quad +\displaystyle \frac{1}{2}\sum \limits _{i=1}^{p}\sum \limits _{j=1}^{p}\beta _{i}\beta _{j}((x_{i}\cdot x_{j})+1)\\ \text{ s.t. }&{}~C_{3}-\sum \limits _{j=p+1}^{p+q}\alpha _{j}-\sum \limits _{j=p+1}^{p+q}\alpha _{j}^{*}\ge 0,\\ &{}\quad -\nu _{2}+\sum \limits _{i=1}^{p}\beta _{i}\ge 0,\\ &{}\quad 0\le \alpha _{j},\alpha _{j}^{*}\le C_{4},~~ j=p+1,\ldots ,p+q,\\ &{}\quad 0\le \beta _{i}\le \frac{1}{p},~~i=1,\ldots ,p.\\ \end{array} \end{aligned}$$

Concisely, (36) is equal to the following QPP:

37 $$\begin{aligned} \begin{array}{ll} &{}\min \limits _{\widehat{\pi }}~~\displaystyle \frac{1}{2}\widehat{\pi }^\text {T}\widehat{\Lambda }\widehat{\pi }\\ &{}\text{ s.t. }~-\nu _{2}+\widehat{\delta }^\text {T}\pi \ge 0,\\ &{}\qquad C_{3}-\widehat{\psi }^\text {T}\widehat{\pi }\ge 0,\\ &{}\qquad 0\le \widehat{\pi }\le \widehat{C},\\ \end{array} \end{aligned}$$

where

$$\begin{aligned} \widehat{\pi }=\bigg ({\alpha _{-}^{*}}^\text {T},\alpha _{-}^\text {T},\beta _{+}^\text {T}\bigg )^\text {T},\\ \widehat{\delta }=\bigg (0e_{-}^{\text {T}},0e_{-}^{\text {T}},e_{+}^{\text {T}}\bigg )^\text {T},\\ \widehat{\psi }=\bigg (e_{-}^\text {T},e_{-}^\text {T},0e_{+}^\text {T}\bigg )^\text {T},\\ \widehat{C}=\bigg (C_{4}e_{-}^\text {T},C_{4}e_{-}^\text {T},\frac{1}{p}e_{+}^\text {T}\bigg )^\text {T}, \end{aligned}$$

and

$$\begin{aligned} \begin{aligned}\widehat{\Lambda }&=\left( \begin{array}{ll} \widehat{\Lambda }_{1} &{} \widehat{\Lambda }_{2} \\ \widehat{\Lambda }_{2}^\text {T} &{} \widehat{\Lambda }_{3} \\ \end{array} \right) ,\\ \widehat{\Lambda }_{1}&=\left( \begin{array}{ll} ~~~~BB^\text {T}+e_{-}e_{-}^\text {T} &{} -(BB^\text {T}+e_{-}e_{-}^\text {T}) \\ -(BB^\text {T}+e_{-}e_{-}^\text {T})^\text {T} &{} ~~~~BB^\text {T}+e_{-}e_{-}^\text {T} \\ \end{array} \right) ,\\ \widehat{\Lambda }_{2}&=\left( \begin{array}{ll} ~~~BA^\text {T}+e_{-}e_{+}^\text {T} \\ -(BA^\text {T}+e_{-}e_{+}^\text {T})^\text {T} \\ \end{array} \right) , \widehat{\Lambda }_{3}=AA^\text {T}+e_{+}e_{+}^\text {T}. \end{aligned} \end{aligned}$$

By solving the dual problems of (12) and (13), then according to the KKT condition we can get ($\omega _{+}^{*},b_{+}^{*}$) and ($\omega _{-}^{*},b_{-}^{*}$). The new input point $x\in R^{n}$ can be determined by its distance from the positive and negative hyperplane. The decision function is defined as follows:

38 $$\begin{aligned} Class=\arg \min \limits _{k=-,+}\displaystyle \mid (\omega _{k}\cdot x)+b_{k}\mid , \end{aligned}$$

where $\displaystyle \mid \cdot \mid$ is the perpendicular distance of point *x* from the hyperplanes $(\omega _{k}\cdot x)+b_{k}=0,k=-,+$.

### Nonlinear $\nu$-IMNPSVM

Now, we extend the linear $\nu$-IMNPSVM to the nonlinear case. As NPSVM, we only need to apply the kernel function directly to the above dual problem. For the problem (34), its nonlinear case is as follows:

39 $$\begin{aligned} \begin{array}{ll} \min \limits _{\alpha _{+},\alpha _{+}^{*},\beta _{-}}&{}~~\displaystyle \frac{1}{2}\sum \limits _{i=1}^{p}\sum \limits _{j=1}^{p}(\alpha _{i}^{*}-\alpha _{i})(\alpha _{j}^{*}-\alpha _{j})(K(x_{i}\cdot x_{j})+1)\\ &{}\quad -\sum \limits _{i=1}^{p}\sum \limits _{j=p+1}^{p+q}(\alpha _{i}^{*}-\alpha _{i})\beta _{j}(K(x_{i}\cdot x_{j})+1)\\ &{}\quad +\displaystyle \frac{1}{2}\sum \limits _{i=p+1}^{p+q}\sum \limits _{j=p+1}^{p+q}\beta _{i}\beta _{j}(K(x_{i}\cdot x_{j})+1)\\ \text{ s.t. }&{}~~ C_{1}-\sum \limits _{i=1}^{p}\alpha _{i}-\sum \limits _{i=i}^{p}\alpha _{i}^{*}\ge 0,\\ &{}\quad -\nu _{1}+\sum \limits _{j=p+1}^{p+q}\beta _{j}\ge 0,\\ &{}\quad 0\le \alpha _{i},\alpha _{i}^{*}\le C_{2},~~ i=1,\ldots ,p,\\ &{}\quad 0\le \beta _{j}\le \frac{1}{q},~~j=p+1,\ldots ,p+q,\\ \end{array} \end{aligned}$$

where $K(\cdot ,\cdot )$ is the kernel function. The corresponding constructions are similar to the linear case except that the inner product $(\cdot ,\cdot )$ is taken instead of the kernel function $K(\cdot ,\cdot )$.

For the problem (36), its nonlinear case is given by

40 $$\begin{aligned} \begin{array}{ll} \min \limits _{\alpha _{-},\alpha _{-}^{*},\beta _{+}}&{}~~\displaystyle \frac{1}{2}\sum _{i=p+1}^{p+q}\sum _{j=p+1}^{p+q}(\alpha _{i}^{*}-\alpha _{i})(\alpha _{j}^{*}-\alpha _{j})(K(x_{i}\cdot x_{j})+1)\\ &{}\quad -\sum \limits _{j=1}^{p}\sum \limits _{i=p+1}^{p+q}(\alpha _{i}^{*}-\alpha _{i})\beta _{j}(K(x_{i}\cdot x_{j})+1)\\ &{}\quad +\displaystyle \frac{1}{2}\sum \limits _{i=1}^{p}\sum \limits _{j=1}^{p}\beta _{i}\beta _{j}(K(x_{i}\cdot x_{j})+1)\\ \text{ s.t. }&{}~ C_{3}-\sum \limits _{j=p+1}^{p+q}\alpha _{j}-\sum \limits _{j=p+1}^{p+q}\alpha _{j}^{*}\ge 0,\\ &{}\quad -\nu _{2}+\sum \limits _{i=1}^{p}\beta _{i}\ge 0,\\ &{}\quad 0\le \alpha _{j},\alpha _{j}^{*}\le C_{4},~~ j=p+1,\ldots ,p+q,\\ &{}\quad 0\le \beta _{i}\le \frac{1}{p},~~i=1,\ldots ,p.\\ \end{array} \end{aligned}$$

Now we give the algorithm of $\nu$-IMNPSVM as follows: 
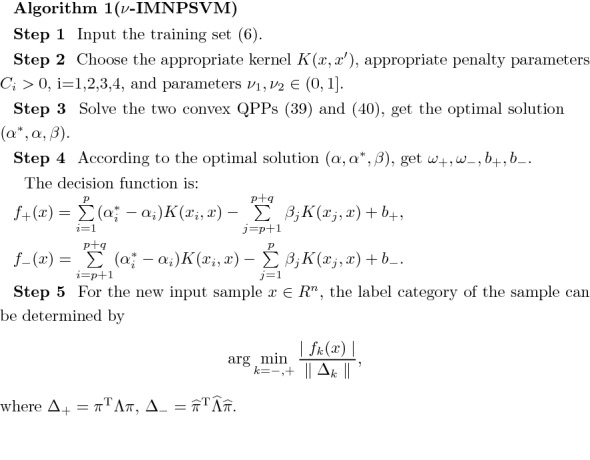


### Significance of the parameter $\nu$

**Figure 2:**
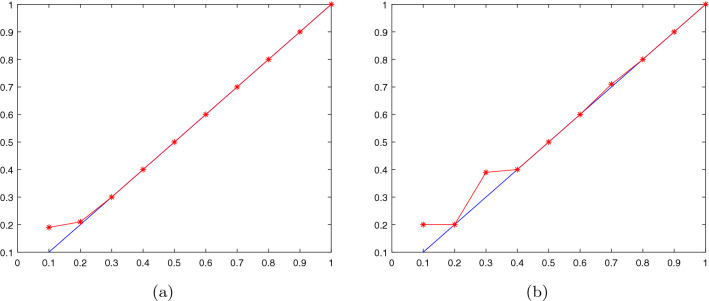
Percentage of SVs change when $\nu _{1}$, $\nu _{2}$ vary in the $\nu$-IMNPSVM two problems. Figure (**a**) is the percentage of the negative SVs in the first $\nu$-IMNPSVM model (35) corresponding to the parameter $\nu _{1}$, the red line is the real percentage of negative SVs rates, and the blue one is the value of $\nu _{1}$. Figure (**b**) is the percentage of the positive SVs corresponding to the parameter $\nu _{2}$ in the problem (37). (**a**) Negative SVs in the first model, (**b**) positive SVs in the second model.

The argument $\nu$ has real value meaning. If the number of support vectors is *q* and the total number of sample points is *l*, there is always $\frac{q}{l}\ge \nu$. Now we use Fig. 2 to illustrate this change. In the figure, we can clearly see the curve of $\nu$ value changing with the ratio of the number of support vectors to the total number of sample points (SVs). Where the red line represents SVs and the blue line represents the value of the parameter $\nu$. The parameter $\nu$ varies in steps of 0.1 between the range (0,1]. We can see that the blue line is always below the red line, which means that the parameter $\nu$ is the lower bound of the SVs ratio. We can adjust the sparsity of the model by adjusting the value of $\nu$, so as to get better classification effect.

## Experimental results

In this section, in order to better verify the performance of the proposed new model $\nu$-IMNPSVM, we compare it with *C*-SVM, $\nu$-SVM, NPSVM and $\nu$-NPSVM under different data sets. All methods are implemented in MATLAB 2016b^28^ on a PC with an Intel Core I5 processor and 4 GB RAM. All training sample points were normalized before training to make the features between [0,1]. The measurement index is based on the classification accuracy, and the classification accuracy is calculated by the standard tenfold cross verification method. For all of the methods, linear and RBF kernel function $K(x,x')=exp\bigg (\frac{-\Vert x-x'\Vert ^{2}}{\delta }\bigg )$ are used.

### Iris data set

**Figure 3:**
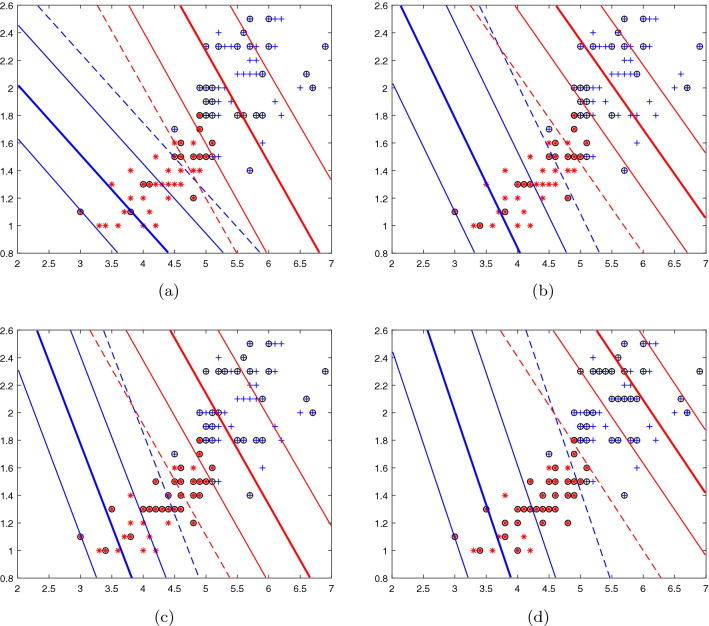
$\nu$-IMNPSVM with the linear kernel: positive proximal line $f_{+}(x)=0$ (red thick solid line), negative proximal line $f_{-}(x)=0$ (blue thick solid line), positive $\varepsilon$-bounded lines $f_{+}(x)=\pm \varepsilon _{+}$ (red thin solid lines), negative $\varepsilon$-bounded lines $f_{-}(x) =\pm \varepsilon _{-}$ (blue thin solid lines), two margin lines $f_{+}(x)=-\rho _{+}$ (red thin dotted line) and $f_{-}(x)=\rho _{-}$ (blue thin dotted line). (**a**) $\nu$=0.1, (**b**) $\nu$=0.3, (**c**) $\nu$=0.5, (**d**) $\nu$=0.7.

First, we validate the model $\nu$-IMNPSVM on the Iris data set^29^, which contains 150 samples corresponding to each row of the dataset. Each row of data contains four characteristics of each sample and the sample category information. It can be divided into three categories (Setosa, Versilcolor and Viginica), here we use only two categories (Versilcolor and Viginica) and two feature (petal length and width of petal). The distribution of data is shown in Fig. 3, where +s and $*$s represent the two categories of Versilcolor and Viginica, respectively.

Here we take the linear kernel as an example and take the parameters separately $C_{1}=C_{3}=20$, $C_{2}=C_{4}=15$, $\nu _{1}=\nu _{2}=\nu$ varies in the range [0.1,0.7] with the step 0.2. Experiment results are shown in Fig. 3, where two proximal lines $f_{+}(x)=0$, $f_{-}(x)=0$, four $\varepsilon$-bounded lines $f_{+}(x)=\pm \varepsilon _{+}$, $f_{-}(x)=\pm \varepsilon _{-}$, and two margin lines $f_{+}(x)=-\rho _{+}$, $f_{-}(x)=\rho _{-}$ are depicted. We can see that the number of support vectors decreases as $\nu$ decreases. And $\nu$ is always the lower bound of the support vector rate while the sparsity keeps increasing.

### UCI data sets

Second, we tested all the methods on several public benchmark sets^29^. All training samples were normalized before training to ensure that the features were between [0,1]. For different data sets, the same number of samples are randomly selected to form balanced data sets, and the above methods are tested respectively.

For all of these methods, the RBF kernel function is $K(x,x')=exp\bigg (\frac{-\Vert x-x'\Vert ^{2}}{\delta }\bigg )$. The parameters $C_{i}, i=1,2,3,4$ are tuned for best classification accuracy in the range $2^{-8}$ to $2^{8}$. The parameters $\nu$, $\nu =\nu _{i}, i=1,2$ are obtained in the range (0,1] with the step 0.1. The experimental process was repeated for 5 times. Table 1 shows the average cross validation results of all methods for different data sets in linear case. Table 2 shows the average cross validation results of all methods for different data sets in nonlinear case. Experimental results show that the classification accuracy of the proposed $\nu$-IMNPSVM model is improved compared with other models.

**Table 1:** Tenfold testing percentage accuracy of linear classifiers.

Data sets	*C*-*SVM*	$\nu$-*SVM*	*NPSVM*	$\nu$-*NPSVM*	$\nu$-*IMNPSVM*
	Accuracy	Accuracy	Accuracy	Accuracy	Accuracy
Australian ($690\times 14$)	85.79 ± 4.85	85.62 ± 3.57	86.64 ± 4.30	86.98 ± 2.13	**87.05 ± 2.20**
BTSC $(748\times 5$)	73.23 ± 4.38	76.25 ± 2.60	76.33 ± 0.28	76.20 ± 0.24	**76.51 ± 0.25**
BUPA liver $(345\times 6$)	74.86 ± 4.53	75.97 ± 4.24	**77.12 ± 4.60**	74.12 ± 2.42	71.01 ± 4.49
CMC $(1473\times 9$)	70.42 ± 4.62	73.02 ± 2.36	74.19 ± 2.25	77.28 ± 1.03	**77.39 ± 0.13**
Diabetes $(768\times 8$)	76.47 ± 2.61	74.85 ± 2.46	**78.78 ± 2.72**	75.23 ± 3.35	75.97 ± 1.56
German $(1000\times 24$)	71.45 ± 2.69	70.04 ± 2.65	74.71 ± 3.13	75.42 ± 2.25	**76.50 ± 2.46**
Haberman $(306\times 3$)	71.45 ± 2.69	73.47 ± 3.26	72.36 ± 4.70	72.58 ± 3.04	**73.53 ± 0.47**
Heart-Statlog $(270\times 13$)	83.36 ± 6.02	85.05 ± 3.20	85.18 ± 5.06	**86.24 ± 2.35**	83.33 ± 6.13
Hepatitis $(155\times 19$)	83.17 ± 4.33	79.84 ± 4.57	85.68 ± 4.19	85.65 ± 1.58	**87.10 ± 3.63**
Ionosphere $(351\times 34$)	89.20 ± 3.45	86.06 ± 3.56	90.15 ± 3.27	90.12 ± 3.14	**90.24 ± 3.22**
Sonar $(208\times 60$)	80.59 ± 4.57	76.24 ± 4.23	73.48 ± 4.45	**82.84 ± 4.28**	81.71 ± 6.01
Spect $(267\times 44$)	76.92 ± 3.18	79.44 ± 3.85	**79.76 ± 3.09**	79.24 ± 2.19	79.40 ± 0.19
WDBC $(569\times 30$)	90.38 ± 4.27	90.24 ± 4.36	92.97 ± 2.16	92.75 ± 6.07	**94.04 ± 2.34**
WPBC $(198\times 34$)	73.25 ± 4.59	76.13 ± 4.27	75.29 ± 4.40	76.27 ± 4.06	**76.92 ± 4.88**

Significant values are in bold.

**Table 2:** Tenfold testing percentage accuracy of nonlinear classifiers.

Data sets	*C*-*SVM*	$\nu$-*SVM*	*NPSVM*	$\nu$-*NPSVM*	$\nu$-*IMNPSVM*
	Accuracy	Accuracy	Accuracy	Accuracy	Accuracy
Australian ($690\times 14$)	84.73 ± 2.60	84.98 ± 5.51	86.51 ± 2.43	86.23 ± 4.29	**88.49 ± 5.33**
BTSC $(748\times 5$)	74.73 ± 3.43	76.12 ± 2.46	75.91 ± 2.24	76.20 ± 2.25	**76.67 ± 1.28**
BUPA liver $(345\times 6$)	75.68 ± 4.50	78.06 ± 4.30	**79.71 ± 4.72**	72.90 ± 4.55	70.61 ± 3.11
CMC $(1473\times 9$)	70.39 ± 4.26	77.37 ± 0.32	75.12 ± 2.65	77.24 ± 1.17	**77.39 ± 0.33**
Diabetes $(768\times 8$)	76.09 ± 2.91	75.10 ± 2.03	**78.01 ± 3.72**	77.32 ± 1.43	75.16 ± 6.05
German $(1000 \times 24$)	74.25 ± 1.49	70.00 ± 0.41	74.71 ± 3.13	76.55 ± 4.49	**77.50 ± 5.90**
Haberman $(306\times 3$)	72.65 ± 2.03	73.53 ± 0.11	73.53 ± 0.48	73.86 ± 2.19	**74.84 ± 2.12**
Heart-Statlog $(270\times 13$)	84.26 ± 4.12	85.05 ± 4.55	83.33 ± 5.24	**87.41 ± 1.81**	83.33 ± 4.11
Hepatitis $(155\times 19$)	81.17 ± 4.62	82.27 ± 4.10	85.90 ± 4.01	85.65 ± 4.24	**86.45 ± 4.03**
Ionosphere $(351\times 34$)	88.84 ± 4.42	84.77 ± 4.22	**96.00 ± 2.46**	91.21 ± 4.67	90.61 ± 3.60
Sonar $(208\times 60$)	83.40 ± 4.57	86.63 ± 3.11	**90.86 ± 5.35**	88.16 ± 2.63	76.19 ± 3.13
Spect $(267\times 44$)	75.44 ± 3.60	78.78 ± 1.82	79.39 ± 0.19	**79.40 ± 0.19**	**79.40 ± 0.38**
WDBC $(569\times 30$)	91.65 ± 2.37	92.11 ± 2.44	**97.89 ± 1.54**	94.74 ± 3.41	93.91 ± 2.36
WPBC $(198\times 34$)	75.36 ± 4.73	75.73 ± 2.62	75.35 ± 5.68	76.27 ± 2.03	**77.28 ± 1.92**

Significant values are in bold.

**Figure 4:**
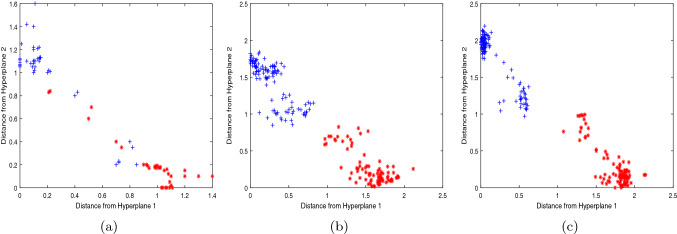
Two-dimensional projections NPSVM, $\nu$-NPSVM and $\nu$-IMNPSVM for 200 test points from the Australian data set. +: scatter plot of the positive point; $*$: scatter plot of the negative point. (**a**) NPSVM, (**b**) $\nu$-NPSVM, (**c**) $\nu$-IMNPSVM.

**Figure 5:**
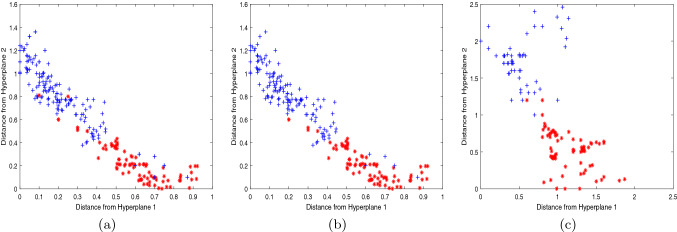
Two-dimensional projections of NPSVM, $\nu$-NPSVM and $\nu$-IMNPSVM for 200 test points from the BUPA liver data set. +: scatter plot of the positive point; $*$: scatter plot of the negative point. (**a**) NPSVM, (**b**) $\nu$-NPSVM, (**c**) $\nu$-IMNPSVM.

**Figure 6:**
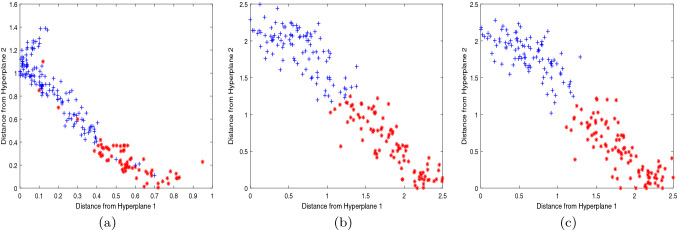
Two-dimensional projections of NPSVM, $\nu$-NPSVM and $\nu$-IMNPSVM for 200 test points from the Heart-Statlog data set. +: scatter plot of the positive point; $*$: scatter plot of the negative point. (**a**) NPSVM, (**b**) $\nu$-NPSVM, (**c**) $\nu$-IMNPSVM.

In order to reflect the experimental effect more clearly, we further compare the 2d scatter plots of NPSVM, $\nu$-NPSVM and $\nu$-IMNPSVM, which were obtained from partial test data points of Australian, BUPA-liver and Heart-Statlog. The data sets we selected were 200 data points randomly selected, including 100 positive and 100 negative points. The scatter plot is obtained by plotting coordinate points based on the vertical distance of the input data point *x* to the two hyperplanes. Figures 4, 5 and 6 depict a comparison of these three methods across three data sets, which can be seen that the classification effect of $\nu$-IMNPSVM is quite obvious.

## Conclusion

In this paper, we propose a new support vector classifier, termed $\nu$-IMNPSVM. It inherits the advantages of NPSVM and further improves the accuracy of data classification on this basis. We replace parameter *C* in NPSVM with parameter $\nu$. The parameter $\nu$ has the following meanings: (1) The parameter $\nu$ balances the two objectives of the support vector machine model, i.e. maximization of interval and minimization of training error. In other words, it minimizes the training error while maximizing the interval. (2) The parameter $\nu$ is different from parameter *C* in existing models and has real value meaning. It represents the upper bound of the ratio between the number of interval error sample points and the total number of sample points in the training set. It also expressed the lower bound of the ratio between the number of support vectors and the total number of sample points. The rate of SVs can be controlled by adjusting the value of parameter $\nu$. (3) In different data sets, the parameter $\nu$ is selected differently, which means that the sparsity of data sets is different. So it can be better applied to the processing of unbalanced data sets. $\nu$-IMNPSVM is different from the model of $\nu$-NPSVM. It only applies the idea of $\nu$-SVM. $\nu$-IMNPSVM retains the free parameter $\varepsilon$ and achieves better classification effect by adjusting the parameters. In addition, the addition of regular terms makes the decision function unique and improves the classification accuracy. However, for large-scale data sets, the computational speed of this model needs to be improved. We hope to get enlightenment from Ref.^26^ and apply the idea of divide-and-conquer to large-scale algorithm in the future. Reasonable and effective solvers should be selected to improve the classification speed. In addition, for support vector regression learning^30^, we hope to get a new idea, which is also under further consideration.

## Data Availability

The datasets analysed during the current study are available in UCI Repository for Machine Learning Databases, https://archive.ics.uci.edu/ml/datasets.php?format=&task=&att=&area=&numAtt=&numIns=&type=&sort=nameUp&view=list. According to the order of data sets in the paper, the corresponding data set numbers on the list view web page are 293, 518, 75, 319, 131, 161, 519, 247, 520, 254, 289, 128, 514, 79 and 81 respectively. The data supporting the findings of the article is available in UCI machine learning repository^29^.
